# Penetration depth of different irrigants in simulated lateral canalsPenetration depth of different irrigants in simulated lateral canals

**DOI:** 10.6026/973206300210797

**Published:** 2025-04-30

**Authors:** Sonali Bansal, Niladri Maiti, Tejal Gowda, Ravi Ranjan Sinha, Sidhartha S.P Behera, Rajshekhar Chatterjee

**Affiliations:** 1 Department of Conservative Dentistry and Endodontics, Kalinga Institute of Dental Sciences, KIIT Deemed to be University, Patia, Bhubaneswar, India; 2 Department of Dentistry, Central Asian University, Uzbekistan; 3 Department of Conservative Dentistry and Endodontics, Dayanand Sagar College of Dental Sciences, Bengaluru, Karnataka, India; 4 Department of Prosthodontics Crown and Bridge, Oxford Dental College, Bengaluru, Karnataka, India; 5 Department of Prosthodontics, S.B Patil Institute for Dental Sciences and Research, Bidar, Karnataka, India; 6 Department of Conservative Dentistry and Endodontics, Gurunanak Institution of Dental Science and Research, Kolkata, West Bengal, India

**Keywords:** Lateral canals, irrigants penetration, sodium hypochlorite, chlorhexidine, ethylenediaminetetraacetic acid (EDTA), root canal disinfection

## Abstract

Proper root canal disinfection needs lateral canals to receive irrigant penetration so that microbial biofilms can be eliminated. Therefore, it is of interest to investigate using confocal laser scanning microscopy how deep NaOCl (5.25%) and CHX (2%) and EDTA (17%) solution penetrated into 60 extracted teeth. NaOCl demonstrated the longest penetration depth at 750 ± 50 µm and trialed EDTA at 620 ± 40 µm and CHX with 480 ± 35 µm penetration depths. Better diffusion is seen in the coronal section (p < 0.05). Thus, NaOCl is the best irrigating agent for disinfecting lateral canals.

## Background:

Complete treatment of the entire root canal system needs both deep cleaning and disinfection of main canals and their complex structure features which include lateral canals and isthmuses and dentinal tubules [1, 2]. Persistent infection and treatment failure becomes likely when lateral canals remain undistincted and uncleaned [3, 4]. The successful outcome of endodontics requires careful penetration of irrigant since mechanical instrumentation fails to eliminate bacteria or debris from affected areas [5]. Different solutions for irrigation have been developed to remove organic and inorganic materials as they simultaneously fight infection-causing microorganisms. Scientists favour sodium hypochlorite (NaOCl) because this solution kills microorganisms effectively while it liquefies organic substances [6]. Irrigants penetration into lateral canals depends on solution concentration together with the amount that is introduced and the technique used for delivery [7]. EDTA works together with NaOCl as an effective solution to eliminate smeared tissue while promoting better irrigants delivery into dentinal tubules and lateral canal structures [8]. The major antimicrobial properties of Chlorhexidine (CHX) as an irrigant are balanced against its minimal tissue-dissolving ability which impacts its penetration within lateral canals [9]. Irrigant penetration efficiency depends on the morphological characteristics of the canal and apex dimension and on the methods used to activate the fluids such as sonic and ultrasonic vibration [10, 11]. Therefore, it is of interest to assess the penetration depth of different irrigants in simulated lateral canals.

## Materials and Methods:

For the investigation 60 human teeth obtained through extraction with mature apices were collected. All soft tissue materials were cleaned from the teeth which then received distilled water storage before experimentation. Researchers cut down the teeth from the cementoenamel junction to achieve a uniform root length of 16 millimeters. Instruments belonging to a rotary nickel-titanium file system were used to achieve apical sizing up to 40/.04 at the conclusion of preparation. Instrumentation required 5 mL of 5.25% sodium hypochlorite (NaOCl) irrigation for each canal between different file usages. A 5ml rinse of 17% ethylenediaminetetraacetic acid (EDTA) washed the canals for one minute before the completion with distilled water. The procedure used a fine round bur with 0.1 mm diameter to create lateral canals at the three sections of each root canal which included the coronal, middle, and apical areas. The cleansing operation involved flushing the canals with saline solution.

## Experimental Groups:

The specimens were randomly divided into three groups (n=20) based on the irrigant used:

[1] Group 1: 5.25% Sodium Hypochlorite (NaOCl)

[2] Group 2: 2% Chlorhexidine (CHX)

[3] Group 3: 17% EDTA

The researchers assigned fluorescent dyes to clarify the visibility of the irrigants when observed through a confocal laser scanning microscope. The side-vented needle at 1 mm working length position delivered each irrigants solution through a 30-gauge tip for 30 seconds at a total volume of 5 mL. The roots underwent horizontal sectioning for the three parts including the apical to the middle to the coronal region after completion of the irrigation process. The examinations using confocal laser scanning microscopy measured lateral canal penetration depths with the help of image analysis software from the evaluated sections. Statistical software performed the analysis of the gathered data. The mean penetration depths between irrigants were assessed through one-way ANOVA analysis that followed a post-hoc Tukey test for comparisons. A statistical significance occurred when the p value reached less than 0.05.

## Results:

The examined irrigants showed substantial variations in how deeply they penetrated through the experimental specimens. The studies showed that the penetration of Sodium hypochlorite (NaOCl) exceeded EDTA and chlorhexidine (CHX). The coronal section of the teeth showed deeper penetrative action for all irrigants in comparison to the middle and apical sections in every experimental group. Table 1 contains details about how the irrigants depths penetrated different root canal levels with micrometric measurements. NaOCl penetrated most deeply into each region at 750 ± 50 µm yet EDTA penetrated to 620 ± 40 µm and CHX penetrated to 480 ± 35 µm. A one-way ANOVA showed a statistically significant difference among the groups (p < 0.05). A post-hoc Tukey test revealed that NaOCl had significantly higher penetration than both EDTA and CHX (p < 0.05), while EDTA also showed significantly greater penetration than CHX (p < 0.05). Additionally, the penetration depths at different root levels within each irrigant group showed significant variation. The coronal third had the greatest penetration, followed by the middle and apical thirds, with a statistically significant difference in all groups (p < 0.05) (Table 2). These results indicate that NaOCl is the most effective irrigant for penetrating lateral canals, followed by EDTA, while CHX demonstrated the least penetration.

## Discussion:

Complete disinfection during root canal treatment requires effective penetration of irrigants into lateral canals. Both the study results confirmed that NaOCl achieved better penetration than EDTA and CHX among the tested solution group. NaOCl achieved superior tissue-dissolution properties that helped it penetrate deeper into lateral canals according to these findings. Data shows that NaOCl achieves better canal penetration because its tissue-dissolving properties enable it to enter sophisticated canal forms [1, 2]. Numerous investigations indicate that NaOCl penetration surpasses other irrigants because of its low surface tension combined with effective fluid movement properties [3, 4]. Currently NaOCl stands as a critical irrigant because it efficiently reaches lateral canals while eliminating biofilms during endodontic treatment [5]. The antiseptic agent EDTA reached the second deepest penetration level and functions to remove smear layers while maximizing dentinal tubule permeability according to literature [6]. The findings on EDTA's penetration level from this study match prior reports that this agent successfully enables irrigant penetration yet demonstrates limited antimicrobial effects [7, 8]. When used with NaOCl EDTA acts as an enhancer to disinfectant penetration by opening up dentinal tubules [9]. Chlorhexidine (CHX) demonstrated the most limited penetration due to its inability to digest tissue structures therefore it penetrated less deep than NaOCl and EDTA [10]. The antimicrobial strength of chlorhexidine is strong and it's substantively performance is good but its failure to dissolve organic substances restricts its penetration abilities into lateral canals [11, 12].

Research evidence shows that CHX has more viscosity than NaOCl which restricts its ability to penetrate complex canal structures [13]. The study revealed that irrigant penetration depth exhibited different patterns through root regions since the coronal section achieved deepest penetration followed by presence in the middle and final coverage in the apical segment. Previous studies have noted that caring fluid reaches further in the coronal section than in the middle or apical section [14]. The apical structure poses higher obstacles to irrigant flow because its limited internal space creates difficulties for solution penetration to reach lateral canals efficiently [15]. Various irrigant activation techniques have been systematically reviewed for their effectiveness in enhancing sodium hypochlorite penetration into root canal systems, highlighting the potential of passive ultrasonic irrigation (PUI) in improving disinfection efficacy [16]. Passive ultrasonic irrigation significantly increases the penetration depth of 2% chlorhexidine digluconate into root dentinal tubules compared to conventional syringe irrigation, as demonstrated using confocal laser scanning microscopy [17]. Both sodium hypochlorite and chlorhexidine, when applied with passive ultrasonic irrigation, exhibit similar depths and percentages of penetration into dentinal tubules, suggesting PUI's effectiveness in enhancing irrigant delivery [18]. The penetration of irrigants depends on three major criteria: delivery methods as well as activation techniques and the selected final irrigation protocol. Scientific research demonstrates that passive ultrasonic irrigation and sonic activation optimize irrigant penetration in lateral canals because they produce acoustic streaming and cavitation effects [3, 5]. The research would benefit from studies that assess the relationship between different activation techniques and irrigant penetration together with their clinical effects.

## Conclusion:

NaOCl stands as the most efficient irrigant for reaching deep into lateral canals thus reinforcing its crucial role in endodontic disinfection. Endodontic irrigation becomes more effective through the addition of NaOCl together with EDTA to remove the smear layer. However, it is necessary to evaluate alternative activation methods and various irrigant concentrations in order to find optimal endodontic irrigation practices.

## Figures and Tables

**Table 1:** Mean penetration depth (µm) of different irrigants in lateral canals

**Irrigant**	**Coronal Third (Mean ± SD)**	**Middle Third (Mean ± SD)**	**Apical Third (Mean ± SD)**	**Overall (Mean ± SD)**
NaOCl (5.25%)	900 ± 40	750 ± 50	600 ± 45	750 ± 50
EDTA (17%)	800 ± 35	610 ± 42	450 ± 38	620 ± 40
CHX (2%)	650 ± 30	470 ± 32	320 ± 28	480 ± 35
Comparison of mean penetration depth of irrigants at different root levels.

**Table 2:** Comparison of penetration depths at different root levels within each irrigant group

**Root Level**	**NaOCl (Mean ± SD)**	**EDTA (Mean ± SD)**	**CHX (Mean ± SD)**	**p-value**
Coronal Third	900 ± 40	800 ± 35	650 ± 30	< 0.05
Middle Third	750 ± 50	610 ± 42	470 ± 32	< 0.05
Apical Third	600 ± 45	450 ± 38	320 ± 28	< 0.05
Statistical comparison of irrigant penetration depth at different root levels.
